# Longitudinal Associations of Self-Reported Visual, Hearing, and Dual Sensory Difficulties With Symptoms of Depression Among Older Adults in the United States

**DOI:** 10.3389/fnins.2022.786244

**Published:** 2022-01-27

**Authors:** Olivia J. Killeen, Xiaoling Xiang, Danielle Powell, Nicholas S. Reed, Jennifer A. Deal, Bonnielin K. Swenor, Joshua R. Ehrlich

**Affiliations:** ^1^Department of Ophthalmology and Visual Sciences, University of Michigan, Ann Arbor, MI, United States; ^2^Institute for Healthcare Policy and Innovation, University of Michigan, Ann Arbor, MI, United States; ^3^School of Social Work, University of Michigan, Ann Arbor, MI, United States; ^4^Department of Health Policy and Management, Johns Hopkins Bloomberg School of Public Health, Baltimore, MD, United States; ^5^Department of Epidemiology, Johns Hopkins Bloomberg School of Public Health, Baltimore, MD, United States; ^6^Cochlear Center for Hearing and Public Health, Johns Hopkins Bloomberg School of Public Health, Baltimore, MD, United States; ^7^Department of Otolaryngology-Head and Neck Surgery, Johns Hopkins University, Baltimore, MD, United States; ^8^Wilmer Eye Institute, Johns Hopkins University School of Medicine, Baltimore, MD, United States; ^9^Center on Aging and Health, Johns Hopkins University School of Medicine, Baltimore, MD, United States; ^10^Institute for Social Research, University of Michigan, Ann Arbor, MI, United States

**Keywords:** vision, hearing, dual sensory impairment, depression, aging

## Abstract

Evidence conflicts on the association between sensory difficulty and depression. Few studies have examined this association using longitudinal or population-based data. We used data from Rounds 1–9 of the nationally representative National Health and Aging Trends Study to evaluate the longitudinal association between self-reported visual, hearing, and dual sensory difficulties and clinically significant depressive symptoms. Multivariable Cox regression models were used to evaluate the hazard of incident depressive symptoms. Group-based trajectory modeling identified depressive symptom trajectories (DSTs). Multinomial logistic regression was used to examine the association between sensory status and DSTs. A total of 7,593 participants were included: 56.5% were female, 53.0% were 65–74 years old, 19.0% (95% CI 17.9–20.2%) had hearing, 5.6% (4.9–6.4%) had visual, and 3.3% (2.9–3.8%) had dual sensory difficulties at baseline. Hazard ratios for depressive symptoms in those with visual, hearing, and dual sensory difficulties were 1.25 (95% CI 1.00–1.56, *p* = 0.047), 0.98 (95% CI 0.82–1.18, *p* = 0.82), and 1.67 (95% CI 1.29–2.16, *p* < 0.001), respectively, relative to those without sensory difficulty. A model with four trajectory groups best fit the data. Group 1 (35.8% of the sample, 95% CI: 34.1–37.4) had persistently low risk of depressive symptoms; Group 2 (44.8%, 43.4–46.3) had low but increasing risk; Group 3 (7.1%, 6.2–8.3) had moderate risk; and Group 4 (12.4%, 11.5–13.3) had moderate to high risk that increased. Compared to those without sensory difficulties, individuals with each difficulty were significantly more likely to belong to a group other than Group 1. This study reveals associations between sensory difficulties and mental health that can inform public health interventions.

## Introduction

Globally, sensory loss and depression are both leading causes of morbidity and can impact daily life (Mathers and Loncar, 2006). It is estimated that there are more than one billion people worldwide who are blind or visually impaired (Bourne et al., 2021) and 1.6 billion who have hearing loss (Haile et al., 2021). Sensory loss disproportionately affects older adults, with an estimated 75% of blindness and 62% of hearing loss occurring in individuals age 50 years and older (Bourne et al., 2021; Haile et al., 2021). In visual impairment, an estimated 80% of cases could have been prevented or have yet to be addressed (WHO, 2019). Similarly, an estimated 50% of hearing loss could be prevented via public health initiatives, and more can be treated through the use of technologies like hearing aids (WHO, 2021).

Dual sensory difficulty is a self-reported decrease in both hearing and visual functioning (Reed et al., 2021). Dual sensory loss is estimated to impact 1–5% of people 65–69 years old and over 20% of people aged 80 and older (Schneider et al., 2011). Dual sensory loss is associated with decreased quality of life (Tseng et al., 2018; Harithasan et al., 2020), subjective poor health (Harada et al., 2008), reduced functional activity (Harada et al., 2008; Cimarolli and Jopp, 2014), anxiety (Cosh et al., 2018a; Simning et al., 2019), suicidal ideation (Cosh et al., 2019), decreased social engagement (Dammeyer, 2014), cognitive decline (Dammeyer, 2014; Harithasan et al., 2020), delirium (Dammeyer, 2014), and even mortality (Dammeyer, 2014). Depressive symptoms are common in individuals with dual sensory loss (Lupsakko et al., 2002). This may be due to behavioral mechanisms such as withdrawal from social situations because of difficulties communicating or seeing and ensuing isolation and/or due to neural changes (Rutherford et al., 2018). Unlike those with single sensory loss, older adults with dual sensory loss may be at increased risk for depressive symptoms because they are unable to compensate for a single sensory loss with retained vision or hearing (Hovaldt et al., 2018). However, prior studies using varying methodologies have yielded conflicting results on the association between depressive symptoms and hearing, visual, and dual sensory loss (Crews and Campbell, 2004; Sloan et al., 2005; Chou, 2008; Pronk et al., 2011; Carrière et al., 2013; Kiely et al., 2013; Hong et al., 2015; Stam et al., 2016; Zheng et al., 2016; Heesterbeek et al., 2017; Brewster et al., 2018; Cosh et al., 2018b; Han et al., 2019; Simning et al., 2019; Lawrence et al., 2020) as well as between clinical depression and sensory loss (Bernabei et al., 2011; Hsu et al., 2016; Crews et al., 2017). To our knowledge, there are no studies examining the association of hearing, visual, and dual sensory difficulty with depressive symptoms over a period of greater than 2 years in a nationally representative sample of older United States adults. However, such data are vital to the development of public health programs and interventional studies that may seek to address this issue.

Using data from the National Health and Aging Trends Study (NHATS), a nationally representative panel study of Medicare beneficiaries 65 years and older, we hypothesized that older adults with self-reported hearing, visual, and dual sensory difficulties are more likely to develop clinically significant symptoms of depression. We also hypothesized that those with self-reported hearing, visual, and dual sensory difficulties would have worse depressive symptom trajectories over time. A deeper understanding of the complex interplay between sensory difficulties, incident depression, and depressive symptom trajectories may help inform the future development of interventions to promote mental health by optimizing late-life sensory function and creating inclusive environments for older adults with sensory loss.

## Methods

We used data from Round 1 (2011) through Round 9 (2019) of the NHATS public-use datasets^1^. NHATS is a nationally representative panel study of Medicare beneficiaries aged 65 years and older. A total of 7,609 community-dwelling older adults or their proxies completed in-person interviews in Round 1. Annual follow-up interviews were conducted with these participants regardless of residential status. After excluding participants with missing data on both visual and hearing difficulty status at baseline (*N* = 16), the study sample consisted of 7,593 participants from the original NHATS cohort. NHATS was approved by The Johns Hopkins University Institutional Review Board and all participants provided informed consent. The data analysis conducted for this study was deemed not regulated because it consisted of secondary analysis of deidentified, publically available data.

### Depressive Symptoms

The two-item Patient Health Questionnaire for Depression (PHQ-2) was administered annually in NHATS. It asks how often a person experienced “little interest or pleasure in doing things” and “feeling down, depressed or hopeless” over the last month. Responses to each question were recorded on a four-point Likert scale (scored 0–3) with total scores ranging from 0 to 6 and a higher score indicating more depressive symptoms. We used a cut-off score of ≥3 to define clinically significant depressive symptoms (Löwe et al., 2005). This cut-off score has a sensitivity of 0.87 and a specificity of 0.78 for major depressive disorder, and a sensitivity of 0.79 and a specificity of 0.86 for any type of depressive disorder (Löwe et al., 2005).

### Sensory Difficulties

Sensory difficulty status was measured at every survey round. A participant was defined as having self-reported visual difficulty if they reported being blind or unable to see across the street and/or read newspaper print, even with glasses. Self-reported hearing difficulty was indicated by a report of being deaf, use of a hearing aid, or being unable to hear well enough to use the telephone or hold a conversation in a room with the TV or radio playing. We created a four-level categorical variable of sensory difficulty, ranging from the reference group of no difficulty (0) to hearing difficulty only (1), visual difficulty only (2), and dual sensory difficulty (3).

It is important to note that a participant who requires glasses to see across the street or read newspaper print is not defined as visually impaired, while a participant who requires hearing aids is defined as hearing impaired. This distinction is made since refractive errors can be fully corrected with glasses or contact lenses. In contrast, hearing aids do not restore normal hearing and require training and behavioral modifications for maximum benefit. These definitions align with those used in prior research (Frank et al., 2019).

### Covariates

Time-invariant socio-demographic covariates included sex, race/ethnicity, and highest education. Time-varying socio-demographic covariates included age in groups, and a dichotomous indicator of Medicaid-Medicare dual eligibility. Time-varying health and functioning covariates included dementia status and a count of the number of self-reported chronic conditions (hypertension, diabetes, heart disease or heart attack, arthritis, osteoporosis, lung disease, cancer, and stroke). Dementia status was measured using the validated NHATS three-level dementia classification (Kasper et al., 2013) that categorizes a respondent as having probable dementia (report of physician diagnosis of dementia/Alzheimer’s disease or scores ≤1.5 standard deviations below the mean on ≥2 cognitive tests of memory, orientation, and executive function), possible dementia (a score ≤1.5 standard deviations below the mean on 1 cognitive test), or no dementia. We also included a dichotomous indicator of proxy interview, where a proxy familiar with the sample person answered the survey questions on their behalf.

### Data Analysis

Descriptive statistics were performed on baseline sample characteristics, stratified by sensory difficulty status at baseline. Bivariate comparisons were performed using Pearson’s chi-squared test for categorical variables and adjusted Wald test for continuous variables. Baseline survey weights, sampling units, and strata factors were adjusted in the estimates, using Taylor linearization for variance estimation (Wolter, 1985).

### Survival Models

The effective analytical sample size for survival analysis was 6,253, after excluding those with depression at baseline (*N* = 1,211) and missing values on covariates (*N* = 129). The Kaplan-Meier estimator (Kaplan and Meier, 1958) was used to estimate the unadjusted survival function for depression across survey rounds with respondents stratified by baseline sensory difficulty status. Multivariable Cox regression models were used to estimate the time to first incident report of depression by time-varying sensory difficulty status. Those who never reported an event prior to the end of the study, loss to follow-up, or death were censored at the last round when they were interviewed. Models were adjusted for time-invariant covariates, including sex, race/ethnicity, and highest education, as well as time-varying covariates, including age groups, dementia status, number of chronic medical conditions, Medicaid eligibility, and proxy respondent. The proportional hazards assumption was evaluated for primary variables of interest.

We chose to include data from proxy respondents, as excluding proxy observations may have introduced selection bias (Skolarus et al., 2010). Prior research has suggested that adjusting statistically for respondent type can decrease bias from proxy responses (Skolarus et al., 2010; Wolinsky et al., 2012). We also reanalyzed each Cox model after pairwise deletion of data from proxies to evaluate the impact of including proxy responses on our models. Baseline survey weights, sampling units, and strata were adjusted in all estimates, using Taylor linearization for variance estimation.

Because of a possible association between gender and depressive symptoms (Harada et al., 2008; Lyu and Kim, 2018), we conducted a sensitivity analysis to determine whether there was effect measure modification by gender on the association between sensory difficulty status and incident clinically significant depressive symptoms.

### Group-Based Trajectory Models

Depressive symptom trajectories were identified using enhanced group-based trajectory modeling. The effective analytical sample size was 7,478 for group-based trajectory modeling after list-wise deletion of missing values (*N* = 115). Group-based trajectory modeling is a specialized application of finite mixture modeling designed to identify clusters of individuals following similar outcomes over time (Nagin, 2005). Like other methods for estimating developmental trajectories, such as growth curve modeling, basic group-based trajectory modeling approach assumes independence of group membership and attrition (e.g., missing at random). However, assuming data are missing at random may lead to biased estimates of trajectory group size (Haviland et al., 2011), particularly in studies on disability (Zimmer et al., 2012). Given the close relationship between depression and disability and prevalent attrition in longitudinal studies of older adults, we adopted an enhanced group-based trajectory modeling that jointly estimates probabilities of group membership and attrition. Estimated probabilities of dropout are specific to each depressive trajectory group.

Depressive symptom trajectories were estimated using the Proc Traj plug-in (Jones and Nagin, 2007) through an iterative process of adding one trajectory group at a time and changing the polynomial type of time for each group. All-cause attrition (e.g., death or other causes of loss to follow-up) was modeled along with depressive trajectories to account for unequal probabilities of attrition among different depressive trajectory groups. Baseline covariates were included in the estimation of trajectories. The optimal trajectory group number was determined by a combination of four criteria: (a) a comparison of the change in Bayesian Information Criteria (BIC), (b) the average posterior probability of group assignment (≥0.7), (c) group size such that no less than 5% of the study sample are assigned to one trajectory group, and (d) conceptual considerations of group distinctiveness and interpretability (Nagin, 2005). Each participant was assigned to the trajectory group for which they had the highest posterior probabilities of a group membership. Subsequently, multinomial logistic regression was used to examine depressive symptom trajectories as a function of baseline covariates, including the baseline sensory disability status.

All statistical analyses were conducted using Stata/15.1. All models accounted for the complex design of NHATS using survey weights, strata, and sampling unit variables.

## Results

Baseline characteristics of the entire study sample (*N* = 7,593), including those with depression at baseline, are presented in Table 1 as weighted estimates. More than half of the study population were female (56.5%), aged 65–74 years old (53.0%), and non-Hispanic White (80.1%). Self-reported sensory difficulties were common. There were 19.0% (95% CI 17.9–20.2%) with self-reported hearing, 5.6% (95% CI 4.9–6.4%) with self-reported visual, and 3.3% (95% CI 2.9–3.8%) with self-reported dual sensory difficulty. Eight hundred fifty-one participants used hearing aids in round 1.

**TABLE 1:** Weighted sample characteristics by self-reported sensory difficulty status in Round 1 of the National Health and Aging Trends Study.

	No sensory difficulty (*n* = 5,220)	Hearing difficulty only (*n* = 1,533)	Visual difficulty only (*n* = 509)	Dual sensory difficulty (*n* = 331)	All groups (*n* = 7,593)	*p*-value^3^
Sensory difficulty status, % (95% CI)	72.1 (70.7, 73.4)	19.0 (17.9, 20.2)	5.6 (4.9, 6.4)	3.3 (2.9, 3.8)	–	
Age groups, % (95% CI)						<0.001
65–69 years	32.4 (31.2, 33.5)	14.8 (12.6, 17.3)	26.1 (22.0, 30.7)	11.5 (7.5, 17.1)	28.0 (27.0, 29.0)	
70–74 years	27.5 (26.5, 28.6)	20.2 (17.8, 22.9)	16.6 (12.6, 21.6)	11.2 (7.1, 17.2)	25.0 (24.1, 25.8)	
75–79 years	19.0 (17.9, 20.1)	20.5 (18.0, 23.2)	16.8 (13.1, 21.1)	17.4 (13.2, 22.4)	19.1 (18.3, 19.9)	
80–84 years	12.8 (12.0, 13.7)	20.6 (18.6, 22.7)	18.1 (14.9, 21.9)	14.5 (10.7, 19.4)	14.6 (14.0, 15.4)	
85–89 years	6.3 (5.6, 7.0)	15.8 (14.0, 17.6)	14.3 (11.3, 17.9)	24.1 (20.5, 28.1)	9.1 (8.5, 9.8)	
≥90 years	2.1 (1.8, 2.5)	8.2 (7.0, 9.5)	8.1 (6.4, 10.3)	21.4 (17.8, 25.4)	4.2 (3.8, 4.7)	
Sex, % (95% CI)						<0.001
Female	58.2 (56.5, 60.0)	45.9 (43.5, 48.3)	69.5 (64.3, 74.3)	58.9 (53.0, 64.5)	56.5 (55.1, 58.0)	
Male	41.8 (40.0, 43.5)	54.1 (51.7, 56.5)	30.5 (25.7, 35.8)	41.1 (35.5,47.1)	43.5 (42.1, 44.9)	
Race/ethnicity, %						<0.001
White, Non-Hispanic	80.0 (78.1, 81.8)	86.3 (84.1, 88.2)	72.7 (67.8, 77.0)	71.1 (63.7, 77.5)	80.5 (78.7, 82.2)	
Black, Non-Hispanic	8.9 (8.0, 10.0)	3.8 (3.2, 4.6)	13.7 (11.7, 16.0)	6.1 (4.5, 8.2)	8.1 (7.3, 9.0)	
Hispanic	6.5 (5.4, 7.6)	5.3 (4.2, 6.7)	9.6 (6.5, 14.1)	16.5 (10.8 24.2)	4.6 (3.7, 5.8)	
Other	4.6 (3.6, 5.9)	4.6 (3.5, 6.1)	4.0 (2.3, 7.2)	6.4 (3.9, 10.5)	6.8 (5.8, 7.9)	
Education, % (95% CI)						<0.001
Less than high school	19.3 (17.6, 21.2)	23.3 (20.7, 26.1)	33.7 (28.9, 38.9)	46.7 (39.6, 53.8)	21.8 (20.1, 23.6)	
High school	27.3 (25.7, 29.0)	28.5 (26.4, 30.6)	27.3 (23.7, 31.3)	27.5 (22.1, 33.6)	27.6 (26.3, 28.9)	
Some college, no degree	22.2 (20.9, 23.6)	20.0 (17.4, 22.8)	21.9 (17.2, 27.5)	11.4 (7.9, 16.3)	21.4 (20.3, 22.6)	
College graduate	31.1 (28.6, 33.8)	28.2 (25.4, 31.3)	17.1 (13.1, 21.9)	14.4 (10.8, 19.0)	29.2 (27.0, 31.6)	
Dementia status, % (95% CI)						<0.001
No dementia	83.6 (81.4, 85.1)	74.3 (71.2, 77.2)	59.8 (54.1, 65.3)	43.2 (36.2, 50.4)	79.2 (77.5, 80.8)	
Possible dementia	9.8 (8.5, 11.2)	13.7 (11.9, 15.7)	13.9 (10.5, 18.2)	16.1 (11.3, 22.5)	10.9 (9.7, 12.3)	
Probable dementia	6.7 (5.9, 7.5)	12.0 (10.1, 14.2)	26.3 (22.2, 30.9)	40.7 (34.1, 47.6)	9.9 (9.2, 10.7)	
Medicare-Medicaid enrollees, % (95% CI)	10.5 (9.3, 11.8)	11.5 (9.5, 13.9)	23.7 (19.7, 28.3)	27.1 (21.6, 33.3)	12.0 (10.7, 13.3)	<0.001
Number comorbid conditions^1^, mean (95% CI)	2.2 (2.2, 2.3)	2.6 (2.5, 2.7)	3.0 (2.8, 3.2)	3.1 (2.9, 3.3)	2.4 (2.3, 2.4)	<0.001
Proxy respondent, % (95% CI)	3.4 (2.8, 4.1)	6.7 (5.5, 8.2)	17.3 (13.7, 21.6)	30.8 (24.7, 37.8)	5.7 (5.1, 6.4)	<0.001
Clinically significant depressive symptoms^2^, % (95% CI)	12.2 (11.1, 13.3)	15.8 (13.5, 18.3)	26.7 (22.2, 31.7)	39.0 (32.6, 45.9)	14.5 (13.3, 15.8)	<0.001

*^1^Comorbid conditions include hypertension, diabetes, heart disease or heart attack, arthritis, osteoporosis, lung disease, cancer, and stroke.*

*^2^Those with clinically significant depressive symptoms at baseline (N = 1,211) were excluded from Cox regression.*

*^3^Bivariate comparisons were performed using Pearson’s chi-squared test for categorical variables and adjusted Wald test for continuous variables.*

### Survival Analysis

**FIGURE 1:**
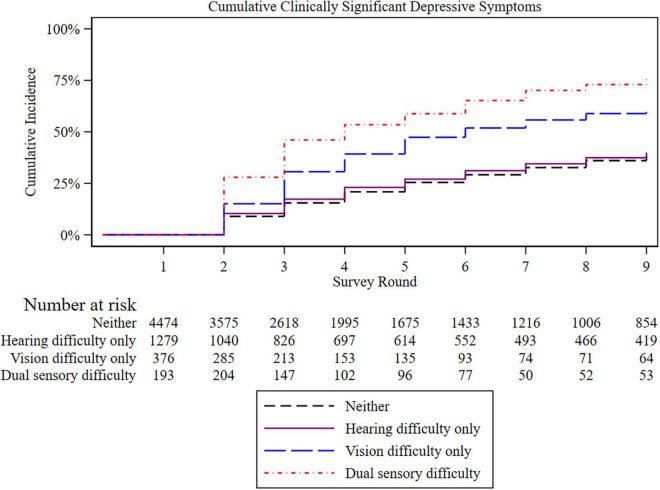
Kaplan-Meier plot of incident cumulative clinically significant depressive symptoms by sensory difficulty. Those with baseline clinically significant depressive symptoms were excluded. Each survey round is separated by 1 year.

The average length of follow-up was 5.3 years in the analytical sample for survival analysis (*N* = 6,253). Figure 1 presents the Kaplan-Meier survival curves depicting the cumulative incidence of clinically significant depressive symptoms by sensory difficulty status. The cumulative incidence of clinically significant depressive symptoms increased over time in all the groups, with the highest incidence among those with dual sensory, followed by visual difficulty.

Results from models estimating the hazard of incident depressive symptoms by sensory difficulty status are presented in Table 2. Compared to those with no sensory difficulty, the hazard ratio (HR) for incident clinically significant depressive symptoms was 1.25 (95% CI 1.00–1.56, *p* = 0.047) for those with visual difficulty and 1.67 (95% CI 1.29–2.16) for those with dual sensory difficulty; there was no increased hazard among those with hearing difficulty only (HR = 0.98, 95% CI 0.82–1.18). We excluded participants requiring a proxy in a sensitivity analysis, and the results were unchanged (Table 2). In a separate sensitivity analysis, we did not detect a main effect for gender or effect modification by gender on the association between sensory difficulty status and the outcome.

**TABLE 2:** Hazard of incident clinically significant depressive symptoms by self-reported sensory difficulty status.

	Hazard ratio^1^	95% confidence interval
**Including proxy (*N* = 6,253)**		
No sensory difficulty	Reference	Reference
Hearing difficulty only	0.98	0.82–1.18
Visual difficulty only	1.25	1.00–1.56
Dual sensory difficulty	1.67	1.29–2.16
**Excluding proxy (*N* = 5,998)**		
Hearing difficulty only	1.01	0.83–1.23
Visual difficulty only	1.28	1.02–1.61
Dual sensory difficulty	1.79	1.34–2.41

*^1^Hazard ratios are adjusted for: sex, race/ethnicity, and highest education, age, dementia status, number of chronic medical conditions, Medicaid eligibility, proxy respondent, and survey round.*

### Group-Based Trajectory Models

**FIGURE 2:**
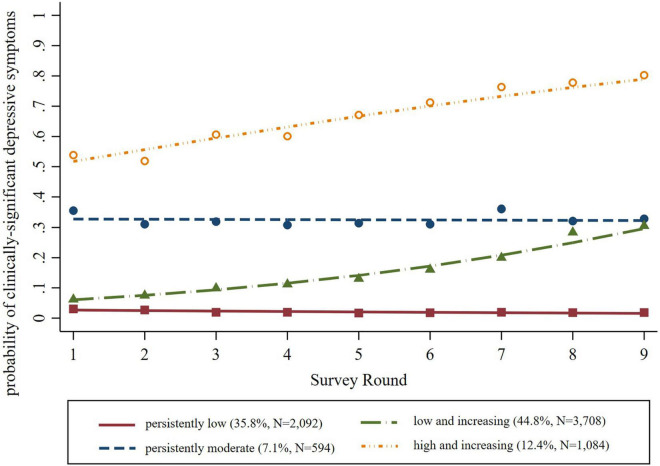
Probability of clinically significant depressive symptoms by trajectory group.

A logit model with four trajectory groups was the best fit for the data based on considerations of changes in BIC (Supplementary Table 1), group distinctiveness and interpretability, group size, and the average posterior probability of group assignment (*p* = 0.86, 0.73, 0.73, and 0.76, for Groups 1–4, respectively). As shown in Figure 2, Group 1 (“persistently low”) represented 35.8% of the weighted sample (95% CI: 34.1–37.4) and had a very low risk of experiencing clinically significant depressive symptoms throughout the 9 year period. Group 2 (“low and increasing”) represented 44.8% of the weighted sample (95% CI: 43.4–46.3) and had a low risk that steadily increased over time. Group 3 (“persistently moderate”) represented 7.1% of the weighted sample (95% CI: 6.2–8.3) and had a moderate risk throughout the 9 year period. Group 4 (“high and increasing”) represented 12.4% of the weighted sample (95% CI: 11.5–13.3) and had moderate to high risk at the start of the study period that steadily increased over time.

Table 3 shows the results of multivariable multinomial logistic regression models predicting depressive trajectory group as a function of sensory difficulty status and other participant-level characteristics at baseline. Relative to those with no sensory difficulty, participants with hearing difficulty had a significantly elevated relative risk ratio (RRR) for membership in the persistently moderate (RRR = 1.46, 95% CI 1.06–2.01) and the high and increasing (RRR = 1.39, 95% CI 1.06–1.81) groups as compared to membership in the persistently low group. Participants with visual difficulty had a significantly elevated RRR for membership in the low and increasing (RRR = 1.69, 95% CI 1.12–2.55), persistently moderate (RRR = 2.01, 95% CI 1.05–3.83) and the high and increasing (RRR = 3.45, 95% CI 2.25–5.31) groups as compared to membership in the persistently low group. Finally, participants with dual sensory difficulty had a significantly elevated RRR for membership in the persistently moderate (RRR = 5.47, 95% CI 2.48–12.05) and the high and increasing (RRR = 10.80, 95% CI 5.07–22.98) groups as compared to membership in the persistently low group.

**TABLE 3:** Multivariable multinomial logistic regression predicting depressive symptom trajectory group as a function of sensory difficulty status and other characteristics.

Baseline characteristics	Low and increasing (*N* = 3,708) vs. Persistently low (*N* = 2,092)		Persistently moderate (*N* = 594) vs. Persistently low (*N* = 2,092)		High and increasing (*N* = 1,084) vs. Persistently low (*N* = 2,092)	
	Relative risk ratio	95% confidence interval	Relative risk ratio	95% confidence interval	Relative risk ratio	95% confidence interval
**Sensory difficulty status**						
Not disabled	Reference		Reference		Reference	
Hearing difficulty only	0.89	0.74–1.07	1.46	1.06–2.01	1.39	1.06–1.81
Visual difficulty only	1.69	1.12–2.55	2.01	1.05–3.83	3.45	2.25–5.31
Dual sensory difficulty	1.50	0.72–3.15	5.47	2.48–12.05	10.80	5.07–22.98

## Discussion

In this nationally representative study of United States adults age 65 years and older, we found a significant longitudinal association over 9 years between self-reported visual and dual sensory difficulties, but not hearing difficulties, and incident clinically significant depressive symptoms. We also found that all sensory difficulties (hearing only, visual only, and dual sensory) were associated with an increased risk of belonging to trajectory groups with a higher baseline risk and an increased risk over time of clinically significant depressive symptoms.

There are several mechanisms by which sensory difficulties could contribute to depressive symptoms. Older adults may withdraw from situations in which they have trouble communicating, hearing, or seeing, leading to isolation (Rutherford et al., 2018). Hearing, visual and dual sensory loss have all been associated with decreased social support and loneliness (Mick et al., 2018). There may also be neural changes associated with sensory difficulties. For example, chronic hearing loss may contribute to changes in the frontal lobe which alter the regulation of emotion (Rutherford et al., 2018). Finally, discrimination, stigma, and a lack of disability-inclusive environments may contribute to the likelihood of depressive symptoms (Shakarchi et al., 2020).

There are inconsistencies in the literature regarding terminology relating to sensory loss, impairment, difficulty, and disability. We chose to use the phrase “difficulty” to reflect the NHATS survey questions about sensory tasks (e.g., ability to hear or see in various scenarios). However, it is important to note that we classified participants as having sensory difficulty if they reported being deaf and/or blind, though these questions relate more closely to identity, a component of disability (Forber-Pratt et al., 2017).

Several studies have examined the cross-sectional and longitudinal associations between dual sensory impairment and depression using nationally representative data. These studies from various global populations have yielded conflicting results. Some cross-sectional studies have found a positive association between dual sensory difficulty and depression (Heine et al., 2019). Several cross-sectional studies that compared the association across sensory difficulties reported the strongest association among those with dual sensory impairment (Hajek and König, 2020; Rong et al., 2020; Pardhan et al., 2021b). In contrast, one study showed a cross-sectional relationship between depression and both visual and dual sensory difficulty but not between hearing difficulty and depression (Chou and Chi, 2004). Another reported a cross-sectional relationship between depression and dual sensory difficulty and hearing difficulty but not visual difficulty (Capella-McDonnall, 2005). Several authors have found gender differences in cross-sectional relationships between sensory impairment and depression (Harada et al., 2008; Lyu and Kim, 2018; Pardhan et al., 2021a). Finally, one cross-sectional study from Malaysia found no association between visual difficulty, hearing difficulty, or dual sensory difficulty and depression (Harithasan et al., 2020), which raises important questions about the role of culture and geographical context in the lived experience of sensory difficulty.

Several longitudinal studies have also investigated this association. Some have reported an association between hearing, visual, and dual sensory difficulty and depressive symptoms, with those with dual sensory difficulty having the strongest association (Han et al., 2019). However, the evidence is conflicting. Other studies have found an association with hearing and dual sensory but not visual difficulty (Kiely et al., 2013); or visual but not dual sensory difficulty (Chou, 2008). Our findings are consistent with population-based longitudinal studies of 4 years of data from China (Xie et al., 2021) and 6 years of data from Norway (Cosh et al., 2018b) that found significant associations between visual and dual sensory, but not hearing difficulty. The magnitude of the associations between visual difficulty (OR = 1.60) and dual sensory difficulty (OR = 1.78) in the Chinese study was similar to the findings in our study (Xie et al., 2021). We build on prior work to present data on the relationship between sensory status and depressive symptoms over a much longer time frame than most prior studies, using 9 years of longitudinal data from a representative sample of United States older adults.

Two prior nationally representative longitudinal studies have investigated dual sensory difficulty and depression in the United States. Capella-McDonnall (2009) analyzed data from the Health and Retirement Study from 1993 to 2006 to investigate changes in depression scores over time in relation to the onset of dual sensory loss. They found a significant increase in depressive symptom scores at the onset of dual sensory loss, as well as a significantly faster increase among that group (Capella-McDonnall, 2009). Since changes in depression scores may not always be clinically relevant, in our study, we used an indicator of clinically significant depressive symptoms that has high sensitivity and specificity for the detection of depressive disorders (Löwe et al., 2005). In another study, Simning et al. (2019) used data from NHATS to examine the odds of clinically significant depressive symptoms cross-sectionally and the odds of persistent symptoms over just 1 year of follow-up. They found that older adults without clinically significant depressive symptoms at baseline who had hearing, visual, or dual sensory difficulty were more likely than those without sensory difficulty to have incident clinically significant depressive symptoms at 1 year (Simning et al., 2019). Our study builds on these findings by estimating the cumulative hazard of depressive symptoms, as well as group-based depressive symptom trajectories over up to 9 years of follow-up.

Our Cox regression models suggest that among those with hearing difficulties, there is a low incidence of clinically significant depressive symptoms. This is consistent with several prior studies (Chou and Chi, 2004; Cosh et al., 2018b; Xie et al., 2021). It is also consistent with our group-based trajectory modeling, which shows that compared to those with no sensory difficulties, individuals with hearing difficulties are not significantly more likely to be in the group with a low and increasing probability of depression compared to the persistently low group. Some have suggested that older adults may adjust to hearing loss by developing new communication skills and utilizing interventions like hearing aids that may decrease the risk of adverse health outcomes like depression (Capella-McDonnall, 2009; Acar et al., 2011; Cosh et al., 2018b). It may also be the case that including participants who use hearing aids in the hearing difficulty group leads to underestimation of the impact of untreated self-reported hearing difficulty on depressive symptoms.

Varying definitions of hearing difficulty across studies may contribute to conflicting findings. Some have defined hearing loss as a self-reported difficulty hearing normal conversation, without specifying whether hearing aids were worn (Cosh et al., 2018b). Others assessed hearing difficulty by asking patients to rate their hearing on a Likert scale either with (Chou and Chi, 2004), or without (Capella-McDonnall, 2005) a hearing aid, or based on whether they typically used a hearing aid (Xie et al., 2021). Still other studies have employed objective hearing measures like pure-tone audiometry (Kiely et al., 2013). Further work is therefore needed to standardize a definition of self-reported hearing difficulty and to understand the factors that shape the association of hearing loss and incident depressive symptomology in hearing aid users and non-users.

In our group-based trajectory models of the probability of clinically significant depressive symptoms, we found four groups of older adults: persistently low, low and increasing, persistently moderate, and high and increasing. Compared to those with no sensory difficulty, individuals with each type of sensory difficulty were significantly more likely to belong to a group with higher baseline risk (e.g., persistently moderate, high, and increasing) and/or increasing risk over time (e.g., low and increasing, high and increasing) versus belonging to the persistently low group.

Several other investigators have examined the association of group-based depressive symptom trajectories in late life with hearing and vision. Schilling et al. (2013) used data over 4 years to describe three group-based trajectories, including a “well-off” pattern of persistently low depressive symptoms, an “adverse stability” pattern of persistently high and stable depressive symptoms, and an “at-risk” pattern of initially mild and increasing depressive symptoms. Good visual functioning was a stronger predictor than independence with activities of daily living of belonging to the “well-off” group. That study did not evaluate auditory functioning. Brewster et al. (2018) analyzed 10 years of data and reported “low probability,” “increasing probability,” and “high probability” trajectories of depressive symptoms; they found that participants with impaired hearing had increased odds of having one of the latter two depressive symptom trajectories; this study did not investigate vision. de la Torre-Luque et al. (2019) analyzed nationally representative data from the United Kingdom collected over 14 years and also found three trajectories, including “low and stable,” “moderate and persistent,” and “high and stable”. In that study, membership in the high and stable group was associated with hearing difficulty, and membership in the moderate and stable group was associated with an increase in visual difficulty over time. That study did not evaluate dual sensory difficulties. These results are similar to ours, though we describe four distinct trajectories of depressive symptoms, analyzed the association of dual sensory difficulty with group-based trajectories, and used data from a large nationally representative United States sample.

This study has several notable strengths. We analyzed data from a nationally representative panel study that provided annual reports of depressive symptoms and sensory difficulty. Participants were surveyed using the same instruments each year, ensuring internal measurement consistency. The PHQ-2 used to assess depressive symptomology in NHATS has high sensitivity and specificity for identifying clinical depression, thus contributing to our study’s clinical relevance (Löwe et al., 2005). Sensory difficulty indicates an individual’s perceived challenges and, in some cases, may be more strongly associated with key late-life health outcomes that objectively measured sensory function (Kuo et al., 2021). Thus, this study using data on self-reported sensory difficulty contributes to a thorough understanding of how sensory factors influence mental health and may complement future studies using data on objectively measured sensory function. Furthermore, by analyzing 9 years of data, this study offers novel conclusions on the long-term association of sensory difficulty and depressive symptoms in older United States adults. To our knowledge, this is the first study to examine the association between hearing, visual, and dual sensory difficulty and group-based depressive trajectories, thus further illuminating the interplay between sensory status and depressive symptoms.

This study also has several limitations. While the PHQ-2 is a validated screening tool, it cannot provide a diagnosis of clinical depression; as a result, we cannot make conclusions about the longitudinal associations between sensory difficulty and clinical diagnoses of depression. Survival bias may have also impacted our results, though, given the elevated risk of death among adults with depression and sensory disabilities, we would expect this to bias our results toward the null hypothesis (e.g., no effect), resulting in an underestimate of the true association. Our definitions of hearing and visual difficulties encompass a wide range of possible functional statuses. Subsequent work should analyze how the severity of sensory difficulty impacts the risk of incident depression. Some prior studies have reported a bidirectional relationship between depression and sensory difficulty (Carrière et al., 2013; Frank et al., 2019; Kim et al., 2020). Future studies should examine the bidirectional association with dual sensory difficulty.

In conclusion, using several distinct modeling approaches, we found that sensory difficulties were associated with depressive symptomology in a nationally representative sample of older United States adults. Recent reports by the *Lancet Global Health* Commission on Global Eye Health and the WHO drew attention to the potential of hearing and vision as levers to promote overall health and well-being (WHO, 2019, 2021; Burton et al., 2021). The current study provides a deeper understanding of the longitudinal associations between vision, hearing, and mental health that may help to inform public health interventions and future interventional research aimed at determining the role of maintaining late-life sensory health for promoting mental health. Such insights are vital to address the needs of a growing population of older adults in the United States and globally.

## Data Availability Statement

Publicly available datasets were analyzed in this study. This data can be found here: https://nhats.org/researcher/data-access.

## Ethics Statement

The studies involving human participants were reviewed and approved by the Johns Hopkins University Institutional Review Board. The patients/participants provided their written informed consent to participate in this study.

## Author Contributions

JE and XX contributed to conception and design of the study. XX performed the statistical analysis. OK wrote the first draft of the manuscript. JE, XX, DP, NR, JD, and BS wrote sections of the manuscript. All authors contributed to manuscript revision, read, and approved the submitted version.

## Conflict of Interest

NR sits on the advisory boards of Shoebox Inc., Good Machine Studio, and Neosensory. JE has consulted for MetLife. The remaining authors declare that the research was conducted in the absence of any commercial or financial relationships that could be construed as a potential conflict of interest.

## Publisher’s Note

All claims expressed in this article are solely those of the authors and do not necessarily represent those of their affiliated organizations, or those of the publisher, the editors and the reviewers. Any product that may be evaluated in this article, or claim that may be made by its manufacturer, is not guaranteed or endorsed by the publisher.

## Footnotes

1
https://www.nhats.org/
