# Supplementary material for: Longitudinal Associations of Self-Reported Visual, Hearing, and Dual Sensory Difficulties With Symptoms of Depression Among Older Adults in the United States
Source: Front Neurosci. 2022 Jan 27;16:786244. doi: 10.3389/fnins.2022.786244 (PMC8829390; doi:10.3389/fnins.2022.786244)
Supplement: Supplementary file 1 [file Table_1.DOCX]

| **Supplementary Table 1.** Tabulated Bayesian Information Criterion (BIC) | | | |
| --- | --- | --- | --- |
| Number of groups | BIC | Null model | 2(∆BIC)^1^ |
| 1 | -20531.79 |  |  |
| 2 | -18644.42 | 1 | 3774.74 |
| 3 | -18573.69 | 2 | 141.46 |
| 4 | -18565.91 | 3 | 15.56 |
| 5 | -18573.06 | 4 | -14.3 |
| 6 | -18586.53 | 5 | -26.94 |
| Note. N = 7,478 for all models.  ^1^ Two times the difference in the BIC between adjacent models | | | |
